# 

**Published:** 1856-03

**Authors:** 


### CONTENTS.

### ORIGINAL COMMUNICATIONS.

PAGE.

ARTICLE I.—Essay on Croup, read before the Atlanta Medical Society, and published by that Body. By EBEN HILLYER, M. D. .... 385

ARTICLE II.—Stramonium as a Remedy in Dysmenorrhœa. By C. W. ASHBY, M. D., Alexandria, Va., .... 392

ARTICLE III.—Extract from the Records of the Atlanta Medical Society. By EBEN HILLYER, M. D., Secretary. Jan. 10. 1856 .... 400

### SELECTIONS.

Additional Observations on the Invariable Existence of a Premonitory Diarrhœa in Cholera .... 404

Average duration of Human Life, .... 408

Law Cases bearing upon the subject of Insanity .... 412

Successful Treatment of Traumatic Tetanus .... 416

On some of the Local and Remote Effects of Chloroform, hitherto unnoticed .. 419

Report on cases of Fever occurring in Guy's Hospital, in 1854 .... 421

Hypnotism in Hysterical Paralysis .... 427

### EDITORIAL AND MISCELLANEOUS.

Atlanta Medical College .... 433

Bibliographical .... 437

Indiscriminate Drugging .... 443

"Stramonium in Dysmenorrhœa" .... 445

Expulsion of Tænia by Pumpkin Seeds .... 446

British Association for the advancement of Medical Science, .... 447

Chloroform in Pneumonia, .... 447

Anæsthetics in the Austrian Army, .... 448

Death of M. Berger, .... 448

To Book Publishers, .... 448

Subscriptions received since last issue, .... 448

